# A Malaria Vaccine That Elicits in Humans Antibodies Able to Kill *Plasmodium* *falciparum*

**DOI:** 10.1371/journal.pmed.0020344

**Published:** 2005-11-08

**Authors:** Pierre Druilhe, François Spertini, Daw Soesoe, Giampietro Corradin, Pedro Mejia, Subhash Singh, Regine Audran, Ahmed Bouzidi, Claude Oeuvray, Christian Roussilhon

**Affiliations:** **1**Biomedical Parasitology Unit, Pasteur Institute, Paris, France,; **2**Division of Immunology and Allergy, Centre Hospitalier Universitaire Vaudois, Lausanne, Switzerland,; **3**Institute of Biochemistry, University of Lausanne, Epalinges, Switzerland,; **4**Sedac Therapeutics, Lille, France; University of LondonUnited Kingdom

## Abstract

**Background:**

*Plasmodium falciparum* merozoite surface protein 3 is a malaria vaccine candidate that was identified, characterised, and developed based on a unique immuno-clinical approach. The vaccine construct was derived from regions fully conserved among various strains and containing B cell epitopes targeted by human antibodies (from malaria-immune adults) that are able to mediate a monocyte-dependent parasite killing effect. The corresponding long synthetic peptide was administered to 36 volunteers, with either alum or Montanide ISA720 as adjuvant.

**Methods and Findings:**

Both formulations induced cellular and humoral immune responses. With alum, the responses lasted up to 12 mo. The vaccine-induced antibodies were predominantly of cytophilic classes, i.e., able to cooperate with effector cells. In vitro*,* the antibodies induced an inhibition of the *P. falciparum* erythrocytic growth in a monocyte-dependent manner, which was in most instances as high as or greater than that induced by natural antibodies from immune African adults. In vivo transfer of the volunteers' sera into *P. falciparum–*infected humanized SCID mice profoundly reduced or abrogated parasitaemia. These inhibitory effects were related to the antibody reactivity with the parasite native protein, which was seen in 60% of the volunteers, and remained in samples taken 12 mo postimmunisation.

**Conclusion:**

This is the first malaria vaccine clinical trial to clearly demonstrate antiparasitic activity by vaccine-induced antibodies by both in vitro and in vivo methods. The results, showing the induction of long-lasting antibodies directed to a fully conserved polypeptide, also challenge current concepts about malaria vaccines, such as unavoidable polymorphism, low antigenicity, and poor induction of immune memory.

## Abstract 2

Functional assays suggest that MSP3-based vaccine elicits strong immune response in human volunteers.

## Introduction

The development of a malaria vaccine holds considerable promise, but has been limited by major conceptual and practical difficulties [1]. In view of the imprecise relevance of animal models [2], we have chosen a path where all critical observations leading to go/no-go decisions in the development process were based, as much as possible, on observations made in *Plasmodium falciparum–*infected individuals[3].

Merozoite surface protein 3 (MSP3) is an antigen identified by a novel approach in which the protection, which could be passively transferred by IgG from protected African adults into naïve, infected individuals [4], was used to identify a mechanism of defence [5,6]. The latter, called antibody-dependent cellular inhibition (ADCI), was used to screen a *P. falciparum* genomic expression library and, eventually, to identify MSP3 as the target of protective antibodies in humans [7]. This is not the way most other vaccine candidates have been selected [1].

The decision to move from preclinical investigations into clinical trials with an MSP3-based vaccine resulted from convergent data from a series of studies that strengthened the association between protection and antibodies directed to this particular antigen, namely, (a) that anti-MSP3 antibodies, either naturally occurring or elicited by immunisation, could achieve parasite killing in the presence of normal monocytes, either in vitro [7,8] or (b) in vivo by passive transfer in *P. falciparum–*infected immunocompromised mice [8,9]; (c) similar results were obtained with a human recombinant anti-MSP3 monoclonal antibody (M. Dziegiel and PD, unpublished data); (d) IgG3 anti-MSP3 antibodies were associated with protection in the two African villages of Dielmo and Ndiop [8,10], and in the village of Oo-do in Asia [11]; (e) IgG3 anti-MSP3 antibodies were associated with an improved prognosis of drug-treated cerebral malaria [10]; and (f) strong protection was induced by MSP3 in Cebidae monkeys against a *P. falciparum* challenge [12]. Additional arguments were that (g) the C terminus of the antigen containing the epitopes targeted by ADCI is fully conserved [13,14]; and (h) all proteic and peptidic formulations tested were immunogenic, and particularly a synthetic polypeptide covering three B cell epitopes targeted by protective antibodies (CO, et al., unpublished data) [8].

We therefore decided to initiate a phase-I clinical trial aimed at assessing safety and immunogenicity, using a preparation of the long synthetic peptide (LSP) formulation derived from the conserved region of MSP3 (MSP3-LSP) produced under Good Manufacturing Practices. The construct included three B cell epitopes and four T-cell epitopes, identified in lymphocytes from exposed populations [8]. The clinical trial was designed as a single site, open, randomised, dose-escalating phase-I study in six groups of six naive European volunteers, with two ajduvants, alum and Montanide.

Besides safety and immunogenicity, which are the object of a separate report [15], the planned trial analysis included a number of biological assays aimed at providing an early indication of the antiparasitic effect of the immune responses induced. This included determinations of antibody reactivity with the parasite protein (and not only the epitopic peptides), duration of the antibody response, isotype profile (particularly with respect to the induction of IgG1 and IgG3 classes that can cooperate with monocytes in the ADCI assay [6]), and, importantly, the biological activity of the antibodies against *P. falciparum* as determined in vitro by ADCI and in vivo by passive transfer in *P. falciparum–*infected SCID mice [9].

The MSP3 vaccine was safe and highly immunogenic [15]. The results of the functional tests of the induced immune responses are reported here.

## Methods

### Clinical Trial

The conditions for production of the MSP3 LSP under Good Manufacturing Practices, quality assurance, quality control, pharmacotoxicity, enrolment of the 36 volunteers, and clinical and laboratory investigations, have been described in detail elsewhere [15]. Briefly, 36 volunteers with no previous history of malaria were recruited for the study and were randomly allocated to six treatment groups, with no major imbalance in sex and age distributions. The study was approved by the Institutional Review Board. Immunisations were performed subcutaneously at months 0, 1, and 4. Samples for immunological studies were taken 1 mo after each immunisation, i.e., at months 1, 2, and 5. The MSP3 LSP polypeptide was adjuvated either by Montanide ISA720 (SEPPIC, Paris, France) or adsorbed onto aluminium hydroxide (alum; Berna Biotech, Bern, Switzerland). The dose-escalating trial design included four dosages with Montanide of 10 μg, 20 μg, 30 μg, or 100 μg of polypeptide, and two groups with alum with either 30 μg or 100 μg of polypeptide. In view of the very strong immunogenicity of the molecule, and of local DTH reactions mostly with Montanide (in four of the five cases recorded), the original protocol of immunisation was amended by the clinicians [15] so that the final regimens received by the six different treatment groups were as follows: for Montanide (each polypeptide dose given in micrograms), 10–10–10, 30–30–10, 100–10–10, and 20–20–20, and for alum, 30–30–30 and 100–10–10. Thirty volunteers underwent the full immunisation schedule. The corresponding 90 sera collected at months 0, 5, and 12 were used in the present study.

### Immunological Analysis

#### Lymphocyte and antibody assays

Immune responses were assessed using as antigen the MSP3-LSP peptide, as well as the four epitopic peptides MSP3a, MSP3b, MSP3c, and MSP3d as described [8,15].

The conditions of lymphocyte and antibody assays have been described in detail [15]. The proliferative responses of the volunteers' lymphocytes were assessed in sextuplicate wells, incubated for 6 d at 37 °C in 5% CO_2_ in the presence of MSP3-LSP at 10 μg/ml and pulsed with 1 μCi of ^3^H-TdR [15]. Results were expressed as stimulation indices calculated as the ratio of the mean ^3^H-thymidine incorporation (cpm) in the presence and the absence of antigen. The interferon gamma (IFN-γ) concentration in pooled supernatants from sextuplicate wells was evaluated by ELISA (Elipair, Diaclone, France), according to the manufacturer's instructions, and expressed as the mean ± standard deviation (SD). Antibodies were determined by ELISA as described [16, 17], using, along with positive and negative controls, test samples diluted 1/100 in microtitre plates (Nunc Maxisorp, Naperville, Illinois, United States) coated with MSP3-LSP at 7 μg/ml in PBS and blocked in PBS with 5% nonfat milk. Results were expressed as arbitrary units defined as the ratio of the test sample OD to the mean OD +3 SDs of 20 individual healthy blood donor samples. In consecutive individual experiments with a set of 40 sera, the results expressed in arbitrary units varied by less than 5% from one experiment to the other. Anti-MSP3-LSP isotypes were determined as described previously [17], and results expressed as individual ratios of pre- and postimmune plasma sample OD. The cytophilicity ratio was calculated as the sum of cytophilic divided by noncytophilic classes ([IgG1 + IgG3]/[IgG2 + IgG4 + IgM]).

#### Reactivity with parasite proteins

Mature schizont-stage proteins of *P. falciparum* (3D7 clone) were extracted in Laemmli sample buffer, submitted to electrophoresis, and transferred to nitrocellulose as described [7]. Nitrocellulose strips were incubated with a 1:100 dilution of each volunteer's serum, washed, and incubated with alkaline phosphatase-labelled anti-human IgG diluted 1/5,000, and the reaction revealed using NBT and BCIP (Promega, Madison, Wisconsin, United States). Each of the three samples (months 0, 5, and 12) from each volunteer were tested simultaneously, and month 5 and month 12 samples were studied side by side with a recombinant human anti-MSP3 antibody (M. Dziegiel, et al., unpublished data) used as positive control. The intensity of reactivity was classified as negative, positive (+), or strongly positive (++).

#### Affinity studies

The binding strength of the MSP3-specific antibodies was evaluated by a modified ELISA protocol carried out in the presence of increasing concentrations of the chaotropic thiocyanate ion NH_4_SCN (0.5 M, 1 M, 1.5 M, 2 M, 3 M, and 4 M). Resistance to increasing thiocyanate stringency conditions has been previously reported to provide an estimate of avidity [16,18,19]. An affinity index (AI) representing the molarity of NH_4_SCN that induced a 50% reduction of the maximal antibody signal in ELISA (obtained in the absence of the chaotropic agent) was used to compare the different sera. Ten sera from the village of Dielmo, Senegal, and ten from the village of Oo-do, Myanmar, were used as positive controls [11,17].

### Biological Antiplasmodial Activity of the Antibodies Elicited by Immunisation

#### In vitro monocyte-mediated effect of antibodies (ADCI assay)

PBMCs were obtained from cytopheresis samples of healthy European blood donors without previous exposure to malaria, isolated by density gradient separation on J PREP (TechGen, Les Ulis, France), aliquoted in decomplemented AB+ serum (Institut Jacques Boy, Reims, France) with 10% DMSO (Sigma-Aldrich, St. Louis, Missouri, United States) at a final concentration of 4 × 10^6^ monocytes/vial, and cryopreserved until use (A. Jafarshad, unpublished data). By relying on cryopreserved monocytes from cytopheresis, we could employ in the present study cells from only three donors, and thereby minimize inter-donor monocyte variability of the assay as compared to previously employed techniques [6,8]. The donors were selected based on low or absent direct effect of the monocyte preparation on *P. falciparum* cultures (≤ 15 %),

Following thawing, cells were diluted 1 in 4 in HBSS without calcium and magnesium (Gibco, San Diego, California, United States) buffered with 20 mM Hepes (Gibco). Viability of monocytes, determined with Trypan blue and by nonspecific esterase (Sigma-Aldrich) staining [6], was usually 90–95%. Cells were added to 96-well flat-bottom culture plates (TPP, Trasadingen, Switzerland) at a rate of 2 × 10^5^ monocytes per well, incubated for 1 h at 37 °C and 5% CO_2_, and washed three times with RPMI, thereby separating nonadherent mononuclear cells from the attached monocytes.

Decomplemented volunteer sera were studied in duplicate wells at a concentration of 1:10 (vol/vol) in complete RPMI medium, together with normal human monocytes and *P. falciparum* (3D7 clone) culture at 2% haematocrit and 0.5–1% starting parasitaemia, in a final volume of 100 μl, as described [6]. Negative and positive controls added to each ADCI culture plate included purified IgG from European blood donors with no history of travel to malaria-endemic areas, and purified IgG from the pool of African sera employed for in vivo passive immunisation [20], both at a concentration of 2 mg/ml (corresponding to about 10% of African serum IgG concentration). At 48 and 72 h of culture, 50 μl of complete RPMI medium was added per well. After 96 h of growth, parasitaemia was determined by microscopic counting of more than 50,000 red blood cells on Giemsa-stained thin smears [6,7]. Final parasitaemia was in the range of 8%–15%. Experiments in which the growth of the parasite was not optimal (≤ 10-fold over the 96-h period) were excluded. Each plate included normal human IgG from non-malaria-exposed French blood donors as a negative control, African hyperimmune pooled IgG (effective in passive transfer experiments in Thai patients) as a positive control [20], and test serum, each with and without monocytes. The specific growth inhibition (SGI) index, which takes into account the potential inhibitory effect of either monocytes alone or serum alone, was calculated as follows: SGI = 1 − [(percentage of parasitaemia with monocytes and test IgG/percentage of parasitaemia with test IgG)/(percentage of parasitaemia with monocytes and normal IgG/percentage of parasitaemia with normal IgG)] × 100. For comparison between several ADCI assays, the SGI obtained with each test serum was expressed as the percentage of the SGI recorded with the African IgG pool included in each plate. Each serum was tested three times in duplicate, and results were expressed as the arithmetic mean of the individual SGI obtained in each experiment. Hence a total of 270 individual ADCI assays were performed. Since the mode of calculation of the SGI can result in a negative value when the test serum has a growth-promoting activity, but also when the monocytes have a growth-promoting effect, without effect of the test serum, negative SGI values are shown as “ADCI non-effective,” i.e., with an SGI = 0 in the results section. The ADCI assays were also performed and the SGI was determined with two other parasite strains, in addition to 3D7 parasite clone, i.e., using the Uganda Palo Alto parasite strain for all sera post immunisation (months 5 and 12) and using the Thai T23 strain for all sera collected at month 5. Based on previous experience, the threshold of positivity of the ADCI assay was considered to be 30% or greater of the positive control.

#### In vivo passive transfer of antibodies in *P. falciparum–*infected SCID mice

The procedure employed was similar to that previously described [9]. It was approved by the ethical committee for animal experiments at the Pasteur Institute. Non-adaptive defences of immunocompromised BXN mice were modulated by reducing the number of tissue macrophages and by controlling the number of blood polymorphonuclear neutrophils using intraperitoneal (IP) injections of 0.2 ml of dichloromethylenediphosphonate (Cl_2_MDP) encapsulated in liposomes, and of 300 μg of the anti-PMN monoclonal antibody NIMP-R14, every 4 d. Synchronized ring forms of clone 3D7 in AB^+^RhD^+^ erythrocytes were injected IP, and uninfected AB^+^RhD^+^ erythrocytes were supplemented in the same manner every 3–4 d. Daily thin blood films drawn from the tail vein were used to monitor the development of parasitaemia. Total human PBMCs were isolated by Ficoll-Hypaque (Sigma) and human monocytes were positively selected with CD 14^+^ magnetic beads (MAC kit, Miltenyi Biotec, Bergisch Gladbach, Germany). Purity was determined by non-specific esterase staining (mean > 98%) and viability was estimated by Trypan blue exclusion (mean > 85%).

This recently developed model remains challenging and time-consuming, and it has a substantial failure rate. Parasitaemia can sometimes resume spontaneously in a proportion of animals; however, this always occurs early, during the first week, usually by days 4–8. In contrast, when the parasitaemia reaches day 10, it persists for weeks or even months, and is interrupted only by the accidental death of the animal or by the administration of effective drugs or antibodies (Text S1) [8,9,21]. For the assessment of volunteer antibodies, when the parasitaemia was found to either increase or remain stable for at least 12 d postinfection, 3 × 10^6^ purified human monocytes were engrafted IP in each mouse. Human monocyte density in mouse blood was monitored by FACS analysis using the anti-CD14 monoclonal antibody, revealed by goat anti-mouse IgG-AlexaFluor 188 (Molecular Probes, Eugene, Oregon, United States), diluted 1/500. The same mouse was sequentially injected IP with either monocytes alone or serum alone, followed by monocytes plus volunteer's sera in a volume of 200 μl injected IP. This amount of human serum results in a ∼40-fold dilution of the volunteer's serum in mouse blood as determined by ELISA titration 24 h postinjection, and catabolism led to a progressive disappearance of the transferred Ab within 7 d.

### Statistical Analysis

Univariate analysis was performed using either nonparametric tests (Mann-Whitney U-test and Spearman rank correlation) or a parametric test (unpaired t-test for comparison of mean values). Fisher's exact test was used for contingency table analysis. Multivariate analysis using regression was done using JMP software from SAS Institute (Cary, North Carolina, United States). For longitudinal analysis of 12-mo samples, the individuals' previous results at 5 mo were taken into account by covariance analysis.

## Results

The safety and tolerance of the vaccine were satisfactory, particularly with alum, and are reported in detail elsewhere [15].

### Induction of Strong T and B Cell Responses

**Figure 1:**
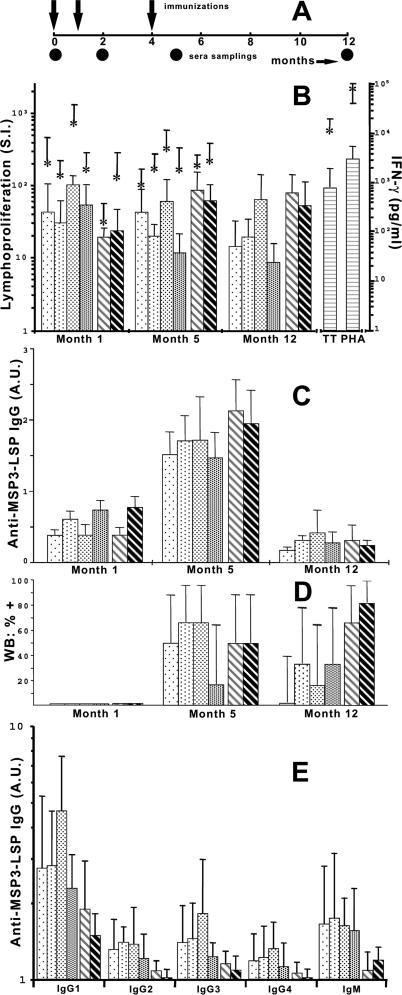
Immunogenicity of the MSP3-LSP in Volunteers Receiving the Vaccine Adjuvated by Montanide or Alum (A) Scheme of immunisation (arrows) and of sampling (plain circles). Samples for immunoassays were taken 1 mo after each immunisation. (B) Lymphoproliferative responses (bars) and IFN-γ secretion (*), ± SD, as compared to controls. PHA, phytohemagglutinin; TT, tetanus toxoid. IFN-γ values for TT and PHA are those obtained using month 5 samples. (C) Mean ELISA IgG titres to the MSP3-LSP at various time points during and after immunisation (months 1, 5, and 12 after the first immunisation). (D) Proportion of WB-positive individuals in each group at different time points ± 95% confidence intervals. (E) Isotype distribution of antibodies measured in ELISA with IgG subclass-specific secondary antibodies (data from samples collected at month 5). In each graph, the increasing grey colour corresponds to increasing immunisation doses, e.g., for Montanide (unhatched bars) from left to right, 10–10–10, 20–20–20, 30–30–10, 100–10–10, and for alum (hatched bars) 30–30–30 and 100–10–10.

Immunogenicity is summarized in Figure 1. The scheme of immunisation and sampling is shown in Figure 1A.

T cell proliferative responses (Figure 1B) were detected in 29 out of 30 volunteers who completed the immunisation schedule, and were as high as with the potent stimulator tetanus toxoid. Stimulation indices were almost maximal from the first immunisation, even at the lowest dose injected, remaining high over the follow-up. Notably, responses were as strong with alum as with Montanide, which contrasts with results obtained in mice and in South American primates, in which alum had been ineffective with this (unpublished data), as with other vaccines [22]. The strongest responses were directed to MSP3 peptides a, b, and c, in the alum groups [15]. Similarly, IFN-γ secretion in response to stimulation by the MSP3-LSP was in the range 10,000–50,000 IU after the first immunisation, as high as that induced by tetanus toxoid (Figure 1B). In contrast, interleukin 4 production was nonsignificant [15].

Antibody responses as measured by ELISA were detected in 77% (23/30) of the volunteers. They increased progressively over time (Figure 1C), despite a moderate decrease between the second and the third injections [15], which was followed by a strong boost. Titres induced by alum-adjuvated MSP3 were lower after the second immunisation than those induced by Montanide [15]; however, they reached similar levels after the third immunisation (Figure 1C).

**Figure 2:**
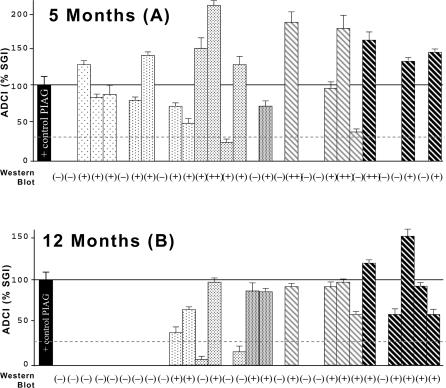
In Vitro Antiparasitic Effect of the Volunteers' Antibodies in ADCI Assay Shown are results obtained with volunteers' serum samples collected either at month 5 (A) or at month 12 (B), as compared to the African immune IgG pool able to transfer clinical protection in humans (dark bars, pool of immune African globulin-positive control). Each bar represents the mean value obtained with each volunteer serum, in three separate experiments ± SD. The results from WB assays (performed with months 5 and 12 samples side by side with a positive control) are shown below those of the ADCI assay for each individual volunteer and are expressed as either negative (−) or positive (+ or ++). For each group, the increasing grey colour corresponds to increasing immunisation doses, e.g., from left to right, Montanide (unhatched bars) 10–10–10, 20–20–20, 30–30–10, and 100–10–10, and for alum (hatched bars) 30–30–30 and 100–10–10. SGI values 30% or greater are considered positive. Dotted line indicates the threshold of positivity of the ADCI assay.

Western blots (WBs) showed the induction of antibodies able to react with the MSP3 48-kDa parasite protein in most but not all volunteer sera [15]. Amongst the ELISA-positive individuals, 22% did not react with the parasite protein by WB, accordingly 78% were positive by WB (Figures 1D and 2). Thus there were qualitative differences among volunteers in the types of antibodies elicited. The overall 60% reactivity to native proteins among immunised volunteers (18/30) indicates that the vaccine formulation is not yet optimal.

Isotype studies revealed that the dominant subclasses were IgG1 followed by IgG3, i.e., the cytophilic antibodies that can cooperate with blood monocytes in the ADCI effect (see Figure 1E). Hence, the antibody isotype balance was very different from that of infected travellers and children from endemic areas, whose responses are predominantly noncytophilic, and was essentially similar to that of adults from endemic areas who have reached a state of protection [10,11,23]. Finally, the isotyping revealed differences in titre between the alum and Montanide groups that were not apparent using anti-total-IgG (see Figure 1C), possibly due to differences in affinity or titre of antibodies directed to each subclass in the anti-IgG.

Epitope mapping revealed other differences in vaccine-induced, as compared to parasite-induced, responses. Whereas natural exposure to the parasite induced antibodies to each of the three B cell epitopes (b, c, and d) included in the LSP [8], LSP immunisation induced antibodies to peptides MSP3c and MSPd, but almost none to MSP3b, except in one volunteer [15]. This may be related to the conformation of the synthetic as compared to the native parasite protein. Noticeably, anti-MSP3c and anti-MSP3d antibodies have been found to be of equal functional importance in defence to those directed to the MSP3b peptide [8].

Affinity, indirectly evaluated by a modified ELISA, indicated that the vaccine adjuvated by alum induced antibodies with significantly higher affinity than those elicited by the Montanide-adjuvated vaccine (mean AI ± 1 SD, 2.03 ± 0.47 and 1.62 ± 0.33, respectively; *p* = 0.0068 by unpaired t-test), and that the affinity increased between the second and third immunisations (AI = 1.64 and 2.03, respectively, in the alum group; *p* = 0.02.). The values obtained by artificial immunisation are of similar magnitude to those observed among protected African or Asian adults (mean AI = 1.64 ± 0.49 in 66 protected individuals in Oo-do) [11].

### Vaccine-Induced Antibodies Have Strong Antiplasmodial Activity

The sera were studied using the bioassays previously found to best reflect protection against malaria in human beings, and which were also at the origin of the identification of the vaccine candidate.

#### In vitro assays

**Figure 3:**
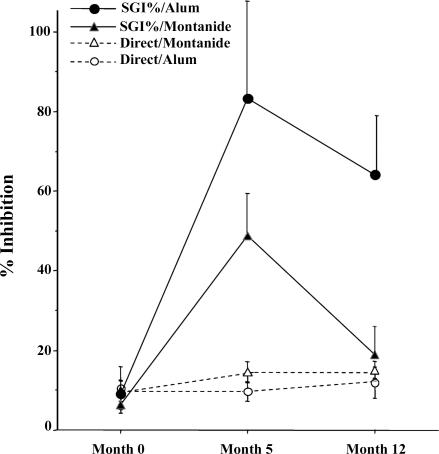
Mean Biological Effect of Antibodies in Either Direct or Monocyte-Dependent Fashion, at Various Time Points with Each Adjuvant Shown are the means ± standard error of the mean of the effects of sera from all volunteers in direct growth inhibition assays (used as a control in each ADCI assay; see Methods) and in monocyte-dependent ADCI assays (sera from 30 volunteers were analyzed at each time point, i.e., the figure summarizes results from 90 sera). Triangles, Montanide-adjuvated vaccine; circles, alum-adjuvated vaccine. Open symbols, direct growth inhibition by antibodies; solid symbols, monocyte-dependent ADCI assays. Months 0, 5, and 12: sera collected before immunisation, 1 mo after the last immunisation, and 12 mo after the first immunisation, respectively.

The antibody-monocyte cooperative effect, as assayed by ADCI (Figure 2A), is expressed as a percentage of the positive control used in each plate—the pool of African IgG able to passively transfer protection in naive recipients [20]. Preimmunisation samples were negative in the assay (SGI ≤ 10%; summarized in Figure 3). MSP3 immunisation elicited antibodies with a parasite-killing effect as strong as that from protected African adults or even stronger in some volunteers, reaching up to two times the potency of African immune IgG (see Figure 2A). Moreover, the few parasites remaining at the end of the ADCI assays performed with the volunteer antibodies were pycnotic, i.e., presumably dead. Alum was at least as effective as Montanide at eliciting antibodies effective at reducing parasite growth. Antiparasite activity correlated with the ability of the antibodies to react with the parasite protein: all but one of the WB-positive sera exerted a profound ADCI effect, i.e., above the threshold value of 30% ADCI (see Figure 2A). The SGI recorded using two other parasite isolates, UPA and FCIP150, were almost identical (the mean SGI values as compared to the parasite clone 3D7 varied by ± 5.1 and 5.4%, respectively,), in agreement with the amino-acid sequence conservation of the antigen among *P. falciparum* isolates. At the serum concentrations employed, no significant direct merozoite invasion inhibitory effect was recorded at any time point (Figure 3).

#### In vivo passive transfer

A subset of nine volunteers' sera were studied individually by passive transfer experiments into immunocompromised mice grafted with human red blood cells in which a sustained *P. falciparum* parasitaemia can be obtained [9]. The effect of total African immune IgG, and of affinity-purified anti-MSP3b and anti-MSP3d antibodies has been documented in this demanding but demonstrative model [8,9]. All types of parasitaemia established for more than 10 d were found previously to be stable, and were interrupted only by administration of antimalarial drugs effective against the parasite strain grafted in mice (but not against drug-resistant parasites) [21] or by ADCI-effective antibodies (but not by ADCI-ineffective antibodies) [8,9], indicating that a decrease in parasitaemia was not accidental, but resulted from these interventions.

**Figure 4:**
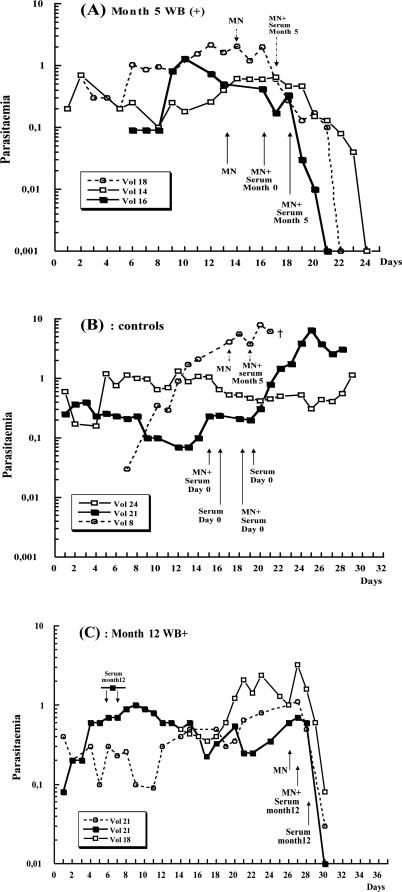
In Vivo Passive Transfer of the Volunteers' Antibodies in *P. falciparum–*Infected Humanised Mice Shown are representative examples of results obtained by passive transfer of Western Blot positive sera collected at month 5 (A), or of control sera (B), and of WB-positive sera collected at month 12 (C). (A) *P. falciparum* infected SCID mice received 200 μl of sera delivered IP from three WB-positive volunteers, collected at month 5, 1 mo after the last immunisation. Shown are results from two mice that received, first, normal monocytes (MN), then monocytes with preimmunisation control sera (month 0), followed by month 5 sera with monocytes (solid arrows corresponding to volunteers 14 and 16, open and solid squares, respectively), one mouse receiving first monocytes followed by monocytes with month 5 serum (dotted arrows, dotted line, open circles) (B) *P. falciparum–*infected SCID mice received 200 μl of sera from controls. Either monocytes followed by monocytes with serum from a WB-negative volunteer (dotted arrows, dotted line, open circles), or monocytes with preimmunisation samples from two volunteers followed by serum alone, repeated twice (plain arrows, solid and open squares). (C) *P. falciparum–*infected SCID mice received 200 μl of sera from three WB-positive volunteers, collected at month 12. All animals received monocytes first, followed by monocytes with the 12-mo serum, followed by serum alone. Reproducibility is shown in two animals receiving the serum from a single donor (volunteer 21, solid squares and open circles). Transfer of serum alone was ineffective (solid squares, days 6 and 7) indicating that the strong in vivo antiparasitic effect depends on monocyte-antibody cooperation.

Mice with steady parasitaemia were injected first with normal monocytes, then a subset with the preimmunisation serum (serum month 0), to ascertain the absence of direct effect of the individual components used in the experiment. This was followed by the injection of the postimmunisation serum (serum month 5). The WB-positive antibodies elicited by immunisation were found to exert a strong parasite-killing effect (Figure 4A). In contrast, preimmunisation samples, as well as sera from WB-negative individuals, were not effective (Figure 4B), even when injected twice (Figure 4B, volunteers 8 and 21), demonstrating the specificity of the in vivo results. Kinetic studies have indicated that the 200 μl of serum injected IP resulted in a ∼40-fold dilution of the volunteers' antibodies in mouse serum. It is remarkable that relatively low concentrations of the antibodies elicited by immunisation could result in the observed profound antiparasite effect. Indeed, the passive transfer of volunteers' antibodies, positive in ADCI and in WB, induced a fast subsequent decrease of the parasitaemia (e.g., Figure 4A, volunteers 14 and 18) and in some animals a full clearance of the parasites (e.g., volunteer 16, not shown in Figure 4A). This contrasts with African IgG, which shows a profound but subtotal effect (i.e., does not clear all parasites), but is in keeping with that of anti-MSP3 purified antibodies [9].

### Results at 12 Months: Long-Lasting Secretion of Antibodies Able to Kill *P. falciparum* in the Alum Group

Analysis of 12-mo samples revealed several important features. T lymphocyte responses remained unusually high in the alum group, as high as after the second or third immunisations, whereas they decreased slightly in the Montanide group (see Figure 1B). ELISA assays of antibodies directed to the synthetic peptide showed an expected drop in titres, particularly in the Montanide group (see Figure 1C), although this decrease did not reach significance. The number of WB-positive individuals also decreased markedly in this group (see Figures 1D and 2B), whereas all WB-positive individuals in the alum group remained positive at 1 y. In addition, possibly due to a maturation of immune responses in relation to the strong T helper (Th) effect, WBs were positive in three individuals that were negative at month 5 (see Figures 1D and 2B). This resulted in a significant difference in the ability to react with the parasite protein between the alum and the Montanide groups (*p* = 0.018). Finally, the mean affinity of antibodies at 1 y also remained higher in the alum group than in the Montanide group (AI = 1.77 and 1.35, respectively).

In agreement with these findings, ADCI assays showed a substantial decrease of the parasite-killing effect of Montanide-induced antibodies as they became unable to react with the native protein (see Figures 2B and 3). Conversely, there was a persistence of a strong ADCI effect in the WB-positive sera from the alum group (see Figure 2B). The relationship of ADCI activity with recognition of parasite native proteins in WB was significant in either the Montanide or alum group (*p* = 0.00021 and 0.041, respectively, by unpaired t-test). Finally, the overall difference in ADCI activity between the Montanide and alum groups was significant (17.2% and 66.3%, respectively; *p* = 0.0016). Therefore, analysis of 1-y samples showed a marked advantage of alum over Montanide, particularly in terms of biological activity of the antibodies.

This was confirmed by in vivo passive transfer experiments of 12-mo serum samples in immunocompromised mice (Figure 4C). Similar results were obtained using three WB-positive sera from the alum group and were ascertained twice (e.g., serum from volunteer 21 in two distinct animals, Figure 4C). Finally, in the absence of human monocytes, the volunteers' antibodies were not effective (control serum 12 mo alone; volunteer 21), in agreement with previous observations with African affinity-purified antibodies [9].

### Antiparasite Activity is a Function of Qualitative and Quantitative Parameters of T and B Cell Responses

Several of the above parameters were significantly linked. Only the main results reaching significance are mentioned here (out of 19 tests carried out). There was a significant association between antibody titres and either affinity (*r* = 0.66, *p* = 0.0002) or cytophilicity ratio (*r* = 0.73, *p* = 0.0005), and this was true for both IgG1 and IgG3 (*r* = 0.92, *p* = 0.0001 and *r* = 0.66, *p* = 0.001, respectively). There was a significant correlation between ADCI activity and the IgG antibody titres (*r* = 0.68, *p* = 0.0001), the cytophilicity ratio (*r* = 0.76, *p* = 0.0001), and cytophilic classes IgG1 and IgG3 (*r* = 0.75, *p* = 0.0001 and *r* = 0.39, *p* = 0.02), as expected for an inhibitory mechanism relying on antibody-monocyte cooperation. T lymphocyte responses were significantly related with IgG levels (*r* = 0.47, *p* = 0.005), antibody affinity (*r* = 0.31, *p* = 0.002), and especially with ADCI antiparasite activity (*r* = 0.41, *p* = 0.0001). IFN-γ production also correlated with antibody levels and ADCI activity (*r* = 0.45, *p* = 0.008 and *r* = 0.37, *p* = 0.03, respectively).

To avoid accidental correlation between two variables, we then performed a multivariate analysis using regression, taking simultaneously into account all available immunological parameters; this analysis showed that the antiparasite ADCI activity was markedly associated with IgG1 levels (*F* ratio = 52.36, *p* < 0.0001) and cytophilicity (*F* ratio = 16.59, *p* = 0.0005), and marginally associated with affinity of antibodies and proliferative T cell responses (*F* ratio = 3.18, *p* = 0.088 and *F* ratio = 3.46, *p* = 0.077, respectively). It is also remarkable that ADCI activity was negatively associated with noncytophilic classes IgG2 + IgG4 (*F* ratio = 3.82, *p* = 0.059) that do not bind to Fc-γ receptors. By multiple regression, an increase of one unit in cytophilicity resulted in a predicted increase of ADCI by 67.5% (*p* = 0.0001), and increase of one unit in affinity resulted in an ADCI increase of 42.9% (*p* = 0.018).

Thus, there was coherence between the immunogenicity of the vaccine in each volunteer, the type of immune response induced and the biological effect of the antibodies elicited.

## Discussion

The phase-I clinical trial conducted with the first MSP3-based vaccine provided the opportunity to assess not only the vaccine's safety and immunogenicity (which were found satisfactory, particularly with alum as adjuvant [15]) but, more importantly, to demonstrate its ability to induce antibodies having a strong anti-parasite effect. This type of immune response, characteristic of immune adults living in malaria-endemic regions, requires under natural conditions 10–15 y of daily exposure to billions of infected red blood cells [4,24,25].

The design of the MSP3-LSP vaccine construct had been based on studies in humans exposed to priming and continuous boosting by parasite molecules. This led to the identification of T cell epitopes and dominant B cell epitopes targeted by cytophilic antibodies that are both associated with exposure-acquired protection and mediate ADCI in vitro [8]. Immunogenicity recorded in human volunteers is in support of this immuno-clinical screening strategy. Indeed, the very good immunogenicity recorded with alum was not predicted by animal studies, since this adjuvant was ineffective in both mice and primates with this (unpublished data) and other antigens [22]. This confirms that experimental models used for preclinical development may not predict faithfully the outcome of immunisations in humans [2].

Cytophilic classes could be readily induced by immunisation with a short vaccine construct, whereas this takes over a decade when the corresponding protein is presented by the intact parasite [11,23]. The correlation with T cell responses suggests that this may relate to an adequate cytokine profile secondary to the strong Th cell response induced by four regions containing Th epitopes. Th1-type activity was obtained, whereas it is currently believed, mostly based on experiments in mice, that alum favours Th2-type responses [26]. The isotype balance may also depend on the characteristics of the three B cell epitopes included in the LSP formulation, which were chosen from six regions in the MSP3 C terminus that are preferentially targeted by cytophilic antibodies [8]. The only difference was that IgG1 predominated over IgG3 in volunteers, whereas it is the reverse in endemic area populations [11,23]. However, the isotype response may differ in populations with distinct genetic backgrounds; that of Europeans is currently not known, as they do not acquire protection through natural exposure. Indeed, the dominant IgG1 heavy chain allotype of Europeans differs from that of Africans, and the former can mediate antibody-dependent cellular cytotoxicity, whereas the latter is less efficient [27–29].

As compared to other phase-I malaria vaccine trials performed with other vaccine candidates, the results obtained with MSP-3 stand favourably. Besides safety, T and B cell responses were within the upper part of the range of those obtained with other candidates. For instance, the 77% responders by ELISA to MSP-3 compare well with the 21% responders to AMA-1 [30]; 16%, 33%, and 16% to MSP-2, MSP1-190L, and RESA, respectively, in combined immunisation [31]; 31%–56% to MSP-1, depending on the vaccine dose [32]; and 72.7%–78% to SPF66 [33,34]. In these studies, investigations were usually limited to total immunoglobulins determined by ELISA and, in one case, immunofluorescence antibody testing, and did not address the isotypic content, the epitope mapping, or the reactivity with native proteins, except for immunofluorescence antibody testing, which is of obvious importance at the time of challenge by the parasite. A major feature of our study is the inclusion of assays aimed at providing an indication of the antiparasitic effect of the induced antibodies. In contrast, reports from previous trials provide no information about this critical feature of the experimental immunisation of volunteers, except for one that found no effect [31].

In contrast, the MSP3-LSP vaccine induced antibodies able to exert a specific parasite-killing effect on *P. falciparum* in two predictive functional bioassays. Both models have been fully validated with respect to clinical findings. The ADCI assay was positive, with African IgG clinically effective upon passive transfer in human recipients; conversely, nonprotective IgG from the recipients, although they contained high titres of antimalarial antibodies, were ineffective in the assay [23]. This assay has been further validated by other research groups [35,36]. The *P. falciparum* mouse model has been more recently developed and remains time-consuming and difficult to employ. Nevertheless, it has been found to mirror drug effects recorded in patients [21], and in passive immunization experiments it reproduced all parasitological events recorded with either protective African IgG or nonprotective control IgG, upon passive transfer in humans [9].

The killing effect correlated with the ability of antibodies to bind to the parasite protein in WBs, which was not obtained in all ELISA-positive volunteers. This is in agreement with current knowledge about the ADCI mechanism, in which the effect is triggered by antibodies binding parasite molecules on the merozoite surface [6]. The killing effect correlated positively with the content of cytophilic classes of antibodies and negatively with noncytophilic classes, in agreement with the monocyte cooperation in ADCI, which requires antibodies binding to monocyte Fc-γ receptors [6,23]. These results provide an improved rationale for further steps of the clinical development of MSP3-based vaccines. It is remarkable that a ∼40-fold dilution of the volunteers' antibodies could achieve in some animals full clearance of a *P. falciparum* parasitaemia. Thus, as compared to IgGs from protected African adults, who have attained the strongest type of protection that can be achieved in humans [25], the biological effects of vaccine-induced antibodies were stronger in some volunteers. Indeed, total African IgG led to a major decrease of parasitaemia but left a few surviving parasites in the *P. falciparum* mouse model [9], consistent with their profound but subtotal effect in Thai recipients [20]. In contrast, anti-MSP3 antibodies elicited by LSP immunisation were, at least in some volunteers, able to sterilize the blood of *P. falciparum–*infected animals. This is reminiscent of the stronger effect previously recorded using anti-MSP3b antibodies, which had a greater effect than African IgGs from which they had been affinity-purified [9]. Similarly, the in vitro effect of antibodies elicited by MSP3 immunisation in the ADCI assay was on average as strong as or stronger than that obtained with African immune IgGs.

This indicates that immunisation by a carefully chosen subunit vaccine can induce antibodies with a greater biological effect than years of exposure to massive amounts of the parasite itself. It therefore raises the hope that MSP3-based vaccines could potentially induce stronger clinical protection than parasite-induced immunisation.

Immune memory has been repeatedly stressed to be a major concern for malarial vaccines [1]. Thus, another remarkable feature of anti-MSP3 responses was their very long duration. In the alum group, high-affinity antibodies reactive with the parasite protein were prevalent over a year. As a consequence, antibodies promoting the ADCI killing effect were still present at 12 mo in the alum group at significant levels. Passive transfer experiments confirmed this long-lasting secretion of antibodies able to profoundly affect *P. falciparum* growth in vivo. This long-term memory is likely to be related to the inclusion of four Th epitopes for human lymphocytes in the LSP, yielding strong T cell responses in 1-y samples. Results suggest an adequate Th-cell driven maturation of B cells, evident in the alum group, leading to low titres of qualitatively improved antibodies. Results in the Montanide group were the opposite, with high initial titres decreasing over time, and in agreement with reports relying on the same adjuvant with other malarial antigens [37,38]. Our analysis reveals a marked advantage of alum over Montanide, which is encouraging, since it is also better tolerated. This challenges the current concept that strong and somewhat toxic adjuvants are unavoidably required, and emphasises the importance of the choice of the antigen.

The vaccine formulation employed here has very attractive biological features, however remains suboptimal. Although strong T cell responses were induced in all volunteers [15], antibodies were not detectable in 23% of them, and antibodies induced in some volunteers failed to react with the parasite protein and reacted with only two of the three B cell epitopes included in the LSP. We had decided in favour of a relatively short synthetic polypeptide vaccine formulation for our first clinical trial, as it is faster to implement than recombinant vaccines. Results support this approach, which led to critical information that could not be gathered in experimental models (e.g., the value of alum, duration of immune responses, reactivity with the parasite protein, and bioactivity of human antibodies). These limitations are likely to be overcome by second-generation, larger recombinants covering the whole C-terminal conserved region, because they will contain more epitopes, and longer polypeptides are usually more immunogenic. It also remains to be seen if the vaccine will be as effective in children from malaria-endemic areas as it was in naive adults.

It has been reported that *P. falciparum,* with part of the gene encoding MSP3 knocked out, could remain viable [39]; this could be a matter of concern for MSP3-based vaccine formulations. However, recent results also show that other genes, such as that encoding MSP6, express epitopes that are fully cross-reactive with MSP3 and are targets of MSP3-induced antibodies, mediating a monocyte-dependent, antibody-dependent, growth-inhibitory effect of equal magnitude to MSP3 epitopes [40]. In this manner, the parasite expresses several identical target antigens to ADCI-mediating antibodies.

The main limitations to malaria vaccine development are currently thought to be high polymorphism in the regions of immunological interest, low antigenicity without the use of powerful and hence toxic adjuvants, short duration of immune responses, and lack of bioassays able to reflect protection and hence to guide preclinical steps [1]. In this initial trial of a MSP3-based vaccine in humans, preliminary results in 30 volunteers indicate that even low doses of MSP3-LSP injected with simple adjuvants readily induced antibodies of cytophilic classes directed to fully conserved epitopes, induced long-lasting effects, and showed strong biological activity against *P. falciparum* erythrocytic stages. Within the limitation of the actual predictive value of these biological assays, which can be confirmed only under Phase II trials, the results indicate that this vaccine can overcome a large number of the identified bottlenecks.

## Supporting Information

Text S1 Present Status of the *P. falciparum* SCID Mouse Model (448 KB PDF).

## Abbreviations

ADCI: antibody-dependent cellular inhibition
AI: affinity index
IFN-γ: interferon-gamma
IP: intraperitoneally
LSP: long synthetic peptide
MSP3: merozoite surface protein 3
SGI: specific growth inhibition
SD: standard deviation
Th: T helper cell
WB: Western blot
