# Supplementary material for: A Malaria Vaccine That Elicits in Humans Antibodies Able to Kill Plasmodium falciparum
Source: PLoS Med. 2005 Nov 8;2(11):e344. doi: 10.1371/journal.pmed.0020344 (PMC1277929; doi:10.1371/journal.pmed.0020344)
Supplement: Supplementary file 1 [file pmed.0020344.sd001.pdf]

P. Druilhe
Bio-Medical Parasitology Unit,
Pasteur Institute, Paris, France.

Present status of the *P. falciparum* scid mouse model

I. Introduction

There is a severe lack of parasite specific tools to study *Plasmodium falciparum* in the laboratory. Over the past years our group has performed experiments aimed at bringing an additional, new, animal model to the very short list of species which can be infected by the main parasite of humans, *P.falciparum*.
When introduced into mice genetically deficient in B-lymphocytes, T-lymphocytres, and/or NK cells functions, intra-erythrocytic blood stages of *P.falciparum* become pycnotic within less than one hour, ie die. Further studies showed that cells of the Monocyte lineage played a major role in defence against *P.falciparum* and indicated that innate, non-adaptive defences of mice against human plasmodia differ from that against Babesia or Theileria pointing to a major role for the macrophage lineage in the host specificity of human plasmodia.
The main difficulty to adress this issue has been that, whereas an animal without B and T lymphocytes can live, an animal without macrophages cannot survive, yet in order to graft *P. falciparum* in mice it is needed to decrease the number of tissue as well as of circulating macrophages by c.a. 60-80%. This can be relatively easily achieved by a number of pharmacological agents which were assesed in systematic manner. However, the parasite is one of the most potent macrophage lineage activator.
Hence the main difficulty, which has been to a large extend solved, has been to find treatment regimens which allowed us to maintain sustained, stable, low numbers of macrophages, despite the conflicting effect of treatments which can destroy them all and that of the parasite which, to the opposite, recruit monocytes and induce their multiplication and fast diferenciation (within hours) into large, very active, macrophages.

II. Reliability of a parasite decrease following administration of a drug or an Ab.

The experiments of in vivo passive transfer of antibodies into the *P.falciparum*/SCID mouse model were designed to avoid to draw conclusions from any accidental drop of parasitemia. Indeed, the mice that are grafted and infected, actually fall into 2 groups. For some of them, the parasitemia resolves spontaneously, and this always happens by day 5, 6 or 7 of the infection (not shown). In contrast, when the parasitemia goes beyond day 8 (Fig. 1-4), it continues in uninterrupted manner for weeks or months (stable parasitemia up to 4 1/2 months has been obtained) and in fact, stands for the duration of the life span of the animal, i.e. is interrupted only by the accidental death of the animal (as the complex scheme of depletion of circulating and tissue macrophages is putting the animals under severe stress and can occasionally induce haemorrhages or anaemia).
In other words, accidental drops of parasitemia are not observed in these animal harboring long-lasting parasitemias (see eg Fig.3 showing a few representative examples of recent studies in animals in which no intervention was made).

In practice, no intervention is done before day 8, as illustrated by figures 1 and 2, taken from our previous papers dealing with immune IgG (2) or with antimalarial drugs (3), respectively.
The correspondance between a given therapeutic intervention, ie a drug or an antibody and the consecutive drop in parasitaemia has been documented in published (Fig 1 and 2)

Fig.1

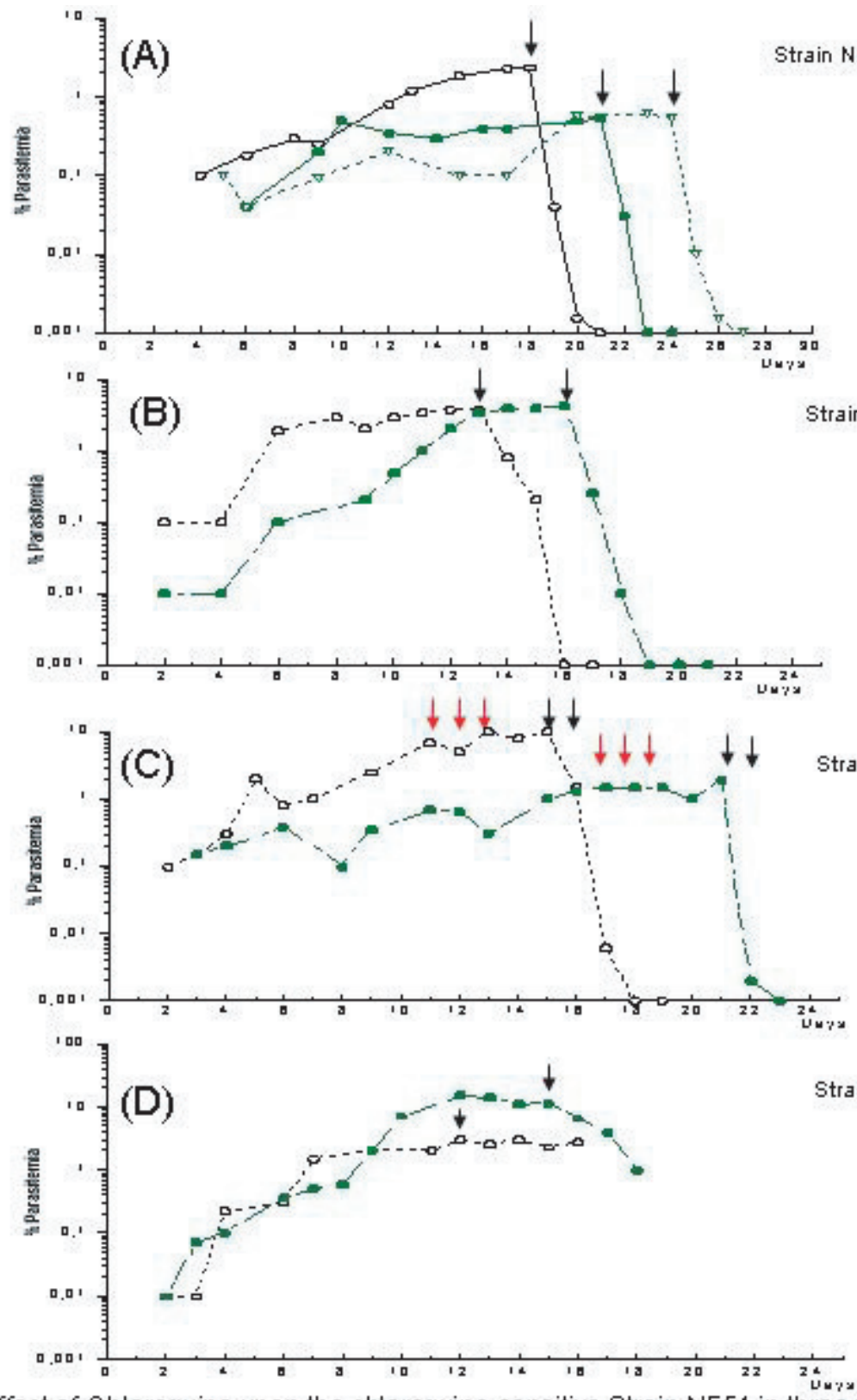

Fig.1:(**A**) Effect of Chloroquine upon the chloroquine-sensitive Strain NF54 in three mice (at a dose of 73 mg/kg for the first 2 days of treatement and 36.5 mg/kg the 3rd day). (**B**) Mefloquine administered to two mice at a dose of 50 mg/kg for 2 days. Strain NF54 Mefloquine-sensitive. (**C**) Effect of Chloroquine (red arrows) upon the chloroquine-resistant Strain T23 in two mice, followed by treatment with dihydroartemisinin (black arrows) at 50 mg/kg for 2 days.. (**D**) Effect of Quinine upon the quinine -resistant Strain T23 (73 mg/kg administered three times a day, for 5 days) in two mice.

and further unpublished experiments (representative examples are provided in Fig 3 and 4)).
Treatments with the anti-malarial drugs chloroquine, quinine, mefloquine, artemisinin, proguanil, inhibitors of phospholipid metabolism, or inhibitors of *P.falciparum* specific enzymes, in immunocompromised mice infected by either drug-sensitive or multi-drug resistant parasites, show that the course of parasite clearance and the morphological effects on the parasite, parallels that seen in infected humans and hence validate its use for drug development. This shows, for instance, that the fastest acting antimalarial drug in humans, artemisin, is also the fastest acting in that model (Fig. 1C), and vice versa for slower acting antimalarial drugs, such as pyrimethamin, or to a leser extent, chloroquine. Parasitaemia established for more than 10 days were interrupted only by administration of antimalarial drugs effective upon the particular parasite strain grafted in mice (see eg . Fig 1 panel A and B), but not upon drug-resistant parasites (see eg . Fig 1 panel C and D). A most demonstrative example is supplied in Fig 1 panels C and D where quinine and chloroquine proved fully uneffective against a Thai isolate highly resistant to those two compounds (IC50 = 1500 nM

and 1200 nM for Chloroquine and Quinine respectively) , whereas the ensuing administration of artemisin was effective (Fig.1C). Examples of further studies using novel antimalarial compounds currently under development are shown in Fig 4 panels A and B, one of which is fully ineffective upon P.yoelii but is effective upon *P.falciparum* in vitro and in the Scid model.

Fig.2

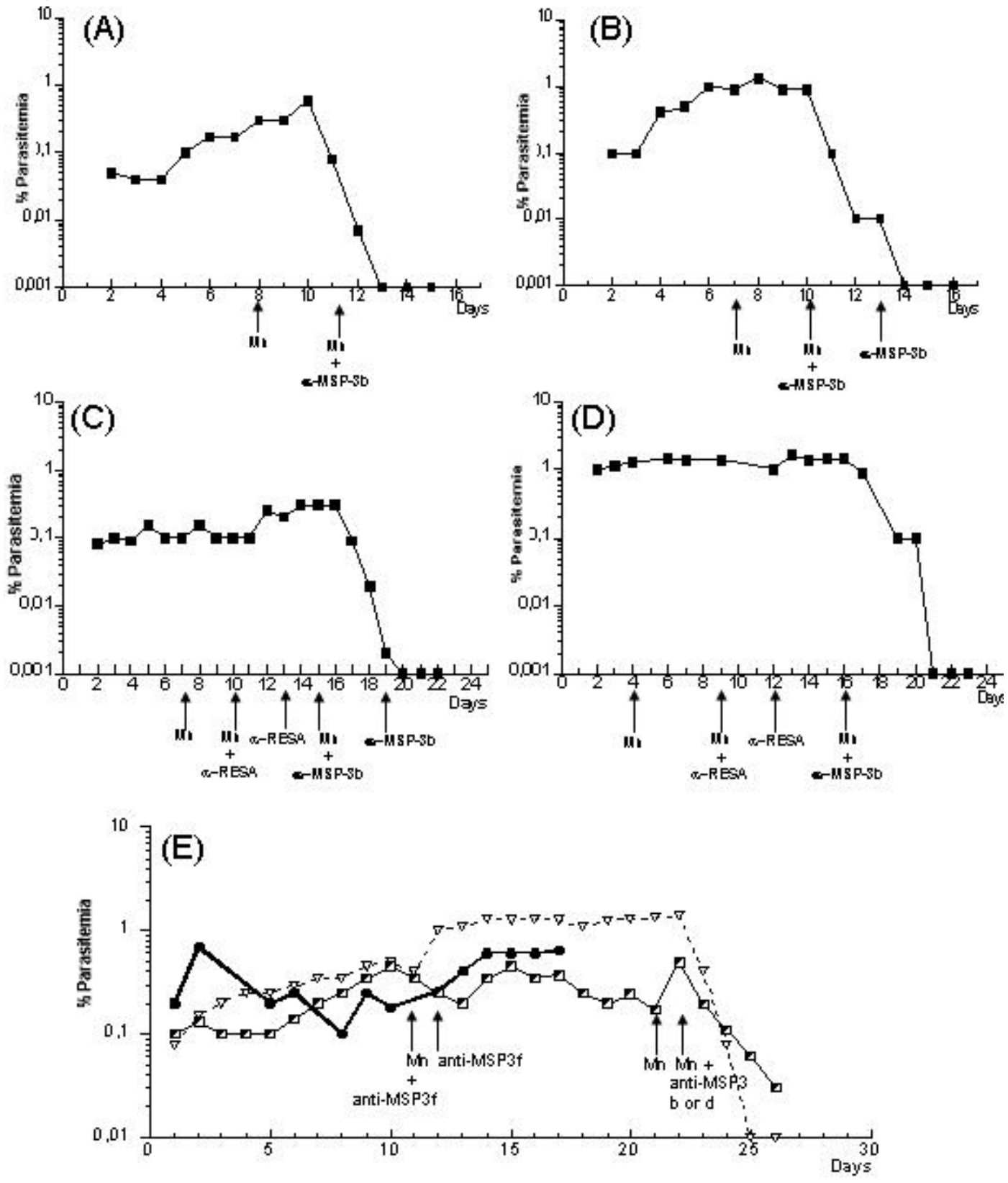

Fig.2:(**A** and **B**) Course of parasitemia in two mice receiving first human monocytes and thereafter the anti-MSP3b Abs together with human monocytes. (**C** and **D**) Two mice receiving sequential treatement first by human monocytes, then by human monocytes together with anti-RESA Abs, then followed by human monocytes together with anti-MSP3b Abs. (**E**) *In vivo* transfer of affinity-purified human anti-MSP3 Abs to peptide b, d or f, together with human monocytes.

Treatments with components of the human immune system yielded particularly important data. In contrast with in vitro studies, the long lasting parasitaemia obtained in Scid allows one to study sequentially the effect of several components of the immune system, eg. Abs specific of various antigens, where each animal constitutes its own control. The improved regulation of physiological parameters under in vivo conditions provide more reliable, less fragile conditions of study as compared to in vitro. Repeated inoculations of clinically active total IgG from African immune adults had no effect upon the course of parasitaemia. Similarly repeated grafting of physiological numbers of leukocytes, either lymphocytes, monocytes, or total PBL had also no parasitological consequence, whereas the combination of both reproducibly resulted in parasite killing in vivo. This demonstrated that the individual components had no non-specific effect effect on the parasitaemia..In the presence of human MN, control European IgG had no whereas African IgG effective upon passive transfer in vivo in humans, was effective (2).

This demonstrating that the effect was related to malaria specific Abs. The model was thereafter applied to the evaluation of vaccine candidates, as a first step of MSP3b, an epitope found to be the target of ADCI mechanism and inducing Abs in humans which are strongly related to the state of acquired protection.

The repeated injection of human monocytes had no effect, as of monocytes and anti RESA Abs, however when this was followed by anti-Msp3b Abs it reproducibly induced parasite clearance at the same speed as that observed with chloroquine (Fig. 2 A, B, C, D).

Further studies using antibodies specific of epitopic peptides " b " , " d ", " f " derived from MSP3 (4) also show the effect upon passive transfer of cytophilic antibodies effective in vitro in the ADCI assay (eg Abs against peptides " b " or " d ") and the absence of effect of anti peptide " f " control Abs, uneffective in vitro in cooperation with Monocytes (Fig 3, panel E). Fig 4 shows more recent studies using either uneffective Abs (eg panel D red arrow) or effective ones (eg panels C and D black arrows)

The fact that the parasitemia drops as a result of treatment ie. the next day with artemisin, in the next two days for quinine or chloroquine (at similar speed as in humans treated), or the next two days with African immune IgG, Abs affinity purified on vaccine candidates, or using immunized volunteers' sera, can hardly be accidental.

Fig.3

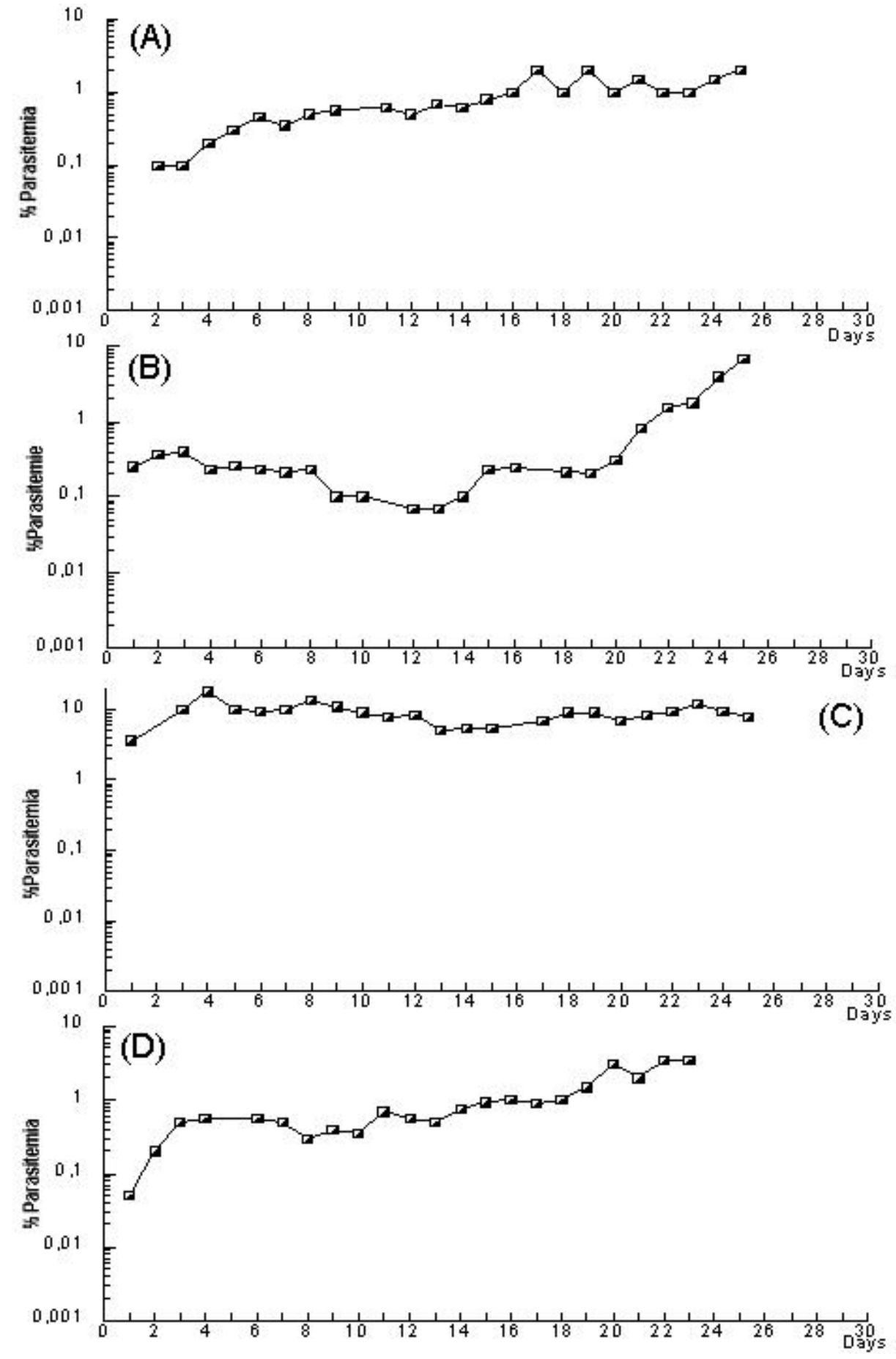

III. Conclusions

Over the past few years, we have shown that it is possible to develop a small laboratory rodent model able to harbour *Plasmodium falciparum* over several weeks and up to 4 months. The model remains today time-consuming, with a substantial drop-out rate. Thus, it has still limitations that restrict its large scale use. However the model can provide at the cost of substantial work, stable *P.falciparum* parasitaemia during which the effects of various interventions, drugs or components of the immune system can be evaluated.

Fig.4

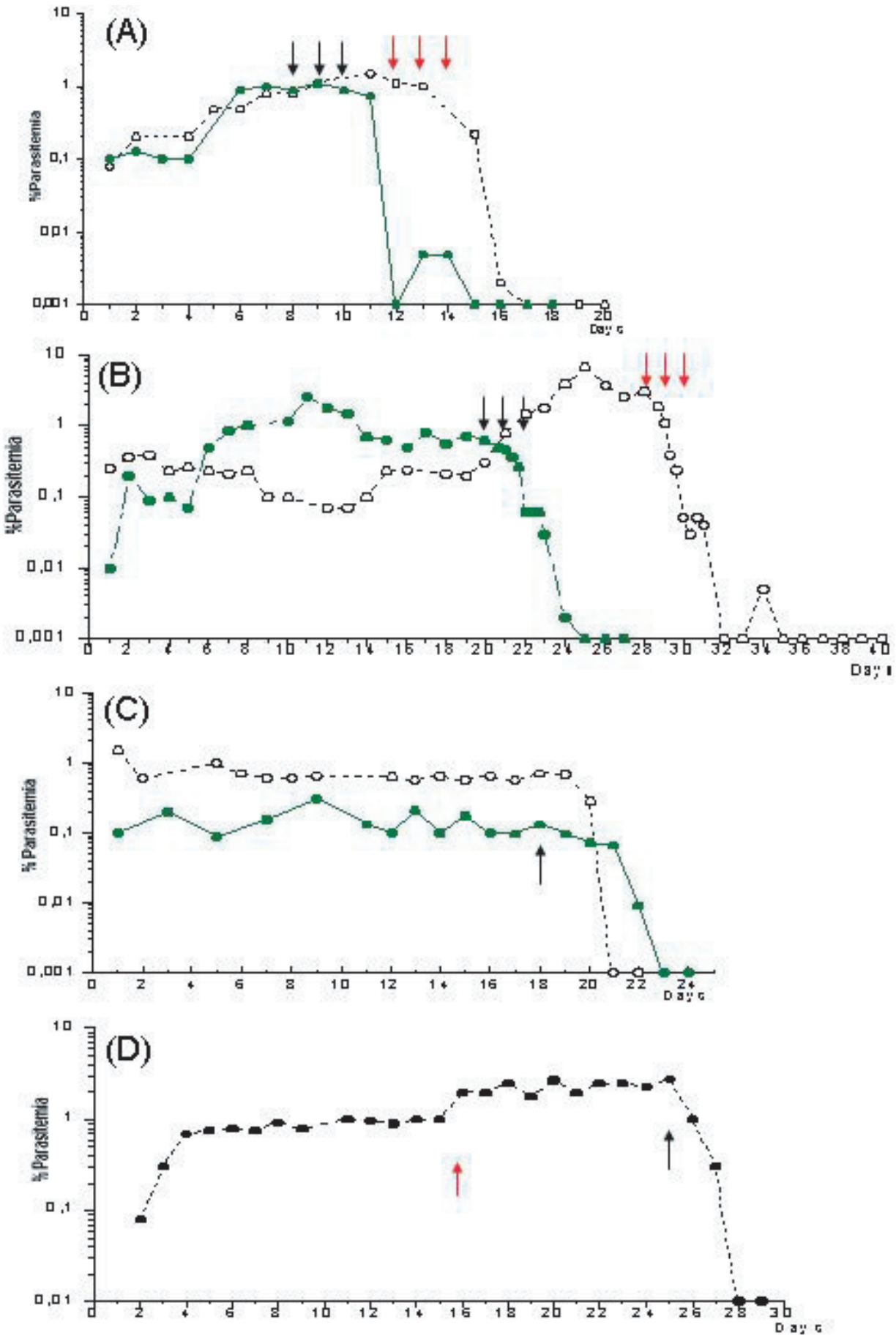

IV. References :

1.BADELL E., PASQUETTO V., VAN ROOIJEN N., DRUILHE P..
A mouse model for human malaria erythrocytic stages.
Parasitol. Today, 1995, 11(6), 235-237.

2. BADELL, E., OEUVRAY, C., MORENO, A, SOE SOE, D., VAN ROOIJEN, N., BOUZIDI, A., DRUILHE, P..
Human malaria in immunocompromised mice: an in vivo model to study defence mechanisms against *P.falciparum*".
J. Exp.Med, 192, 11, 1653-1659

3. MORENO A., BADELL E., VAN ROOIJEN N., DRUILHE P.
Human malaria in immunocompromised mice: a new model for chemotherapy studies
Antimicrobial agents & Chemoth, June 2001, 45, 6, 1847-1853

4. SINGH S, SOE S, MEJIA JP, ROUSSILHON C, THEISEN M, CORRADIN G, DRUILHE P..
Identification of a conserved region of *Plasmodium falciparum* MSP-3 targeted by biologically active antibodies to improve vaccine design.
J Infect Dis. 2004 Sep 1;190(5):1010-8
